# The Role of IL-17 and Th17 Lymphocytes in Autoimmune Diseases

**DOI:** 10.1007/s00005-015-0344-z

**Published:** 2015-06-11

**Authors:** Jacek Tabarkiewicz, Katarzyna Pogoda, Agnieszka Karczmarczyk, Piotr Pozarowski, Krzysztof Giannopoulos

**Affiliations:** Centre for Innovative Research in Medical and Natural Sciences, Medical Faculty, University of Rzeszów, Rzeszow, Poland; Department of Experimental Hematooncology, Medical University of Lublin, Lublin, Poland; Department of Clinical Immunology, Medical University of Lublin, Lublin, Poland

**Keywords:** Th17, IL-17, Autoimmune diseases, Therapy

## Abstract

The end of twentieth century has introduced some changes into T helper (Th) cells division. The identification of the new subpopulation of T helper cells producing IL-17 modified model of Th1–Th2 paradigm and it was named Th17. High abilities to stimulate acute and chronic inflammation made these cells ideal candidate for crucial player in development of autoimmune disorders. Numerous publications based on animal and human models confirmed their pivotal role in pathogenesis of human systemic and organ-specific autoimmune diseases. These findings made Th17 cells and pathways regulating their development and function a good target for therapy. Therapies based on inhibition of Th17-dependent pathways are associated with clinical benefits, but on the other hand are frequently inducing adverse effects. In this review, we attempt to summarize researches focused on the importance of Th17 cells in development of human autoimmune diseases as well as effectiveness of targeting IL-17 and its pathways in pre-clinical and clinical studies.

## Introduction

In the late twentieth century some changes in T helper cell’s classification have been introduced. In 1989 Mosmann and Coffman (1989) described the relationship between functional properties of Th1 and Th2 cells and cytokines produced by them (Mosmann et al. 2005). However, in the nineties of the past century a new cytokine, interleukin (IL)-17, was identified (Rouvier et al. 1993; Yao et al. 1995). Subsequently the presence of a novel Th cell subpopulation (Th17), able to produce IL-17, was revealed.

There are six known isoforms of IL-17, from A to F, but Th17 cells are able to produce only IL-17A and IL-17F (Tesmer et al. 2008). Both of them are pro-inflammatory cytokines. Some researchers have recently shown that IL-17A and/or IL-17F are responsible for development of inflammation in many disorders, especially in autoimmune diseases like rheumatoid arthritis (RA), psoriasis, juvenile idiopathic arthritis (JIA), Crohn’s disease and many others (Adami et al. 2014; Hot and Miossec 2011; Hu et al. 2011; Piper et al. 2014; Tesmer et al. 2008). This special population of CD4^+^ T cells produces also IL-21 (Pelletier and Girard 2007) and IL-22 (Pan et al. 2013b). Both of them are pro-inflammatory cytokines; IL-21 helps to restore the balance between Th17 and Treg cells and IL-22 is a member of IL-10 cytokine family, which is linked to chronic inflammation and participates in pathogenesis of many autoimmune diseases.

Th17, like other Th cells, need specific cytokines and transcription factors for activation and proliferation. Specific molecules regulating Th17 cells functions and properties have become more interesting, after discovery that Th17 cells take part in pathomechanisms of many diseases. Since then researchers have tried to find origin and function of Th17 cells and have been trying to use them in therapy. Today we know two ways of activation of Th17 cells and some factors, which promote and inhibit their differentiation. Th17 cells can be stimulated with the use of IL-6/transforming growth factor (TGF)-β (Ghilardi and Ouyang 2007) or IL-23p40 pathway. Main inhibitors of Th17 cells are cytokines produced by Th1 and Th2 cells, interferon (IFN)-γ and IL-4, respectively (Stumhofer et al. 2007). Noack and Miossec (2014) have described also Th17 reciprocally connections with Treg population. Cai et al. (2012) demonstrated in mouse model, that in exosomes, TGF-β1 delayed inflammatory bowel disease (IBD). At the same time, in lymph nodes, it increased proportion of Foxp3^+^ Tregs and decreased percentage of Th17. The interaction between Th17 and Treg populations is probably very important in pathogenesis of autoimmune diseases, because deviation of critical balance in favor of Th17 cells significantly enhances the severity of disease.

In this review, we describe recent progress in understanding of the involvement of Th17 cells and related cytokines in pathogenesis of some autoimmune diseases. To facilitate finding information about particular disorder each of them was described separately. Finally, we emphasize recent results considering effectiveness of therapies targeting Th17 pathways.

## The Role of Th17 Cells and Related Cytokines in Pathogenesis of Autoimmune Diseases

### Systemic Autoimmune Diseases

Substantial progress in understanding of Th17 development and the effects of IL-17 signaling in immune responses has revealed their potential role in human autoimmune diseases. Systemic autoimmune diseases are in majority characterized by the loss of B cells control, production of autoantibodies, and formation of immune complexes, which contribute to tissue damage.

### Systemic Lupus Erythematosus

Systemic lupus erythematosus (SLE) is a chronic autoimmune disorder that may affect the skin, joints, kidneys, brain, heart, blood and other organs. There is growing evidence in both mouse and human models that IL-17 and Th17 cells play an important role in SLE progression. Increased number of T cells producing IL-17 was found in peripheral blood and inflamed organs of patients with SLE (Crispín et al. 2008; Henriques et al. 2010; Shah et al. 2010). Plasma concentration of IL-17 was increased in new-onset patients and during SLE flares rather than in patients with inactive disease (Chen et al. 2010b; Henriques et al. 2010; Yang et al. 2009). Studies considering involvement of IL-17 in pathogenesis of SLE also proved that concentration of IL-17 correlates with severity of the disease (Chen et al. 2010b; Doreau et al. 2009; Shah et al. 2010). IL-17^+^ T cells were also capable to secrete tumor necrosis factor (TNF), IL-2 and IFN-γ, the cytokines also associated with disease severity (Chen et al. 2010b; Crispín et al. 2008). Doreau et al. (2009) showed that IL-17 alone or in combination with B cell-activating factor controlled the survival and proliferation of B cells and their differentiation into immunoglobulin-secreting cells supporting development of SLE symptoms. Mitoma et al. (2005) demonstrated that decreased expression of IL-21R on B lymphocytes in SLE was significantly associated with nephritis and high-titer anti-double-strand DNA antibodies. Some authors also consider that imbalance between Th17 cells and Th1 or Treg lymphocytes caused by disorders in cytokines regulation could be important in development of SLE. The results of genetic studies suggest that genetic variations and distinct gene expression profiles of cytokines secreted by Th17 cells could contribute to the risk of SLE (Pan et al. 2013a; Yu et al. 2011). Also IL-23, which is one of the main cytokines responsible for development, expansion, and proliferation of Th17 cells, plays an important role in pathogenesis of SLE. Du et al. (2014) showed that the level of IL-23 was elevated in patients with SLE. They also demonstrated the correlation between the increased level of IL-23 and renal disease in SLE. This correlation was also revealed in 2012 by other group of investigators. Kyttaris et al. (2010) observed that IL-23R deficiency prevented the development of nephritis in lupus-prone mice. Therefore, IL-23 is considered to be a potential biomarker for renal involvement in SLE and a potential therapeutic target (Du et al. 2014).

### Rheumatoid Arthritis

Rheumatoid arthritis (RA) is characterized by the chronic inflammation of the synovial membrane. Cell’s interactions induce pro-inflammatory cytokines’ production which in turn activates the mechanisms leading to cartilage and bone destruction. It was confirmed that IL-17 could drive inflammation of joints of patients with RA and could be produced locally in inflamed synovium (Chabaud et al. 1999). Cytokine milieu found within the joints promotes Th17 differentiation, because of high levels of IL-6, IL-12, but not IL-23 and a relatively low abundance of TGF-β (Nistala et al. 2010). Increased serum level of IL-17 was also noticed especially in treatment-naïve patients or patients with systemic symptoms (Liu et al. 2012; Roşu et al. 2012). Interestingly, Arroyo-Villa and coworkers (2012) reported significantly lower percentage of circulating Th17 cells and a lower CD4-derived IL-17 secretion in early RA patients in comparison with healthy controls. Additionally, percentage of circulating Th17 cells negatively correlated with a-CCP titer. On the other hand, they also confirmed that there was no difference between established RA patients and healthy individuals. Additionally, they noticed that decreased number of Th17 lymphocytes in peripheral blood of patients was raised anew after one year of treatment with methorexate. Contrary to these results Zhang et al. (2012) noticed increased percentage of Th17 in patients with the mean disease duration of 8.9 ± 3.9 years. In the well-designed 2-year prospective study Kirkham and colleagues (2006) showed that in RA associated joints’ destruction, IL-17 effects were best seen in patients with shorter disease duration, and IL-1β effects were best pronounced in patients with longer disease duration. Recruitment of Th17 cells to inflamed joint seems to be dependent on expression of CCR6 on these lymphocytes and production of CCL20 by synoviocytes (Hirota et al. 2007). Th17 activity correlates with other laboratory parameters of disease activity as well as with severity of clinical symptoms (Kim et al. 2013; Metawi et al. 2011; Roşu et al. 2012). In the course of RA IL-17 and Th17 cells are not only inflammation inducers but they also activate mechanisms leading to joint destruction. Th17 cells are potent inducers of tissue-destructive enzymes, pannus growth, osteoclastogenesis, angiogenesis (Ito et al. 2011; Moon et al. 2012; Pickens et al. 2010; van Hamburg et al. 2011). The destructive mechanisms influence joint’s tissues and are mediated by different pathways associated with IL-17. Osteoclasts can be differentiated via COX-2-dependent PGE2 synthesis (Ito et al. 2011; Kotake et al. 1999), while fibroblasts activation and proliferation are mediated by mTOR and MAPK p38 signaling (Kehlen et al. 2002; Saxena et al. 2011). The study of Sato et al. (2006) suggests that Th17 cells do not act directly on osteoclast precursor cells, although they expressed RANKL. Induction of osteoclasts is rather associated with osteoclastogenesis-supporting cells, possibly through IL-17-mediated induction of RANKL on osteoblastic cells and dendritic cells (Page and Miossec 2005; Sato et al. 2006). In the context of therapeutic targeting of IL-17 in RA, it seems to be important that Th17 cells act rather TNF independently under arthritic conditions (Koenders et al. 2006). Studies considering influence of anti-TNF treatment on Th17 cells led to inconsistent results. Notley et al. (2008) showed in mouse model of RA that inhibition of TNF with soluble receptor or monoclonal antibodies (mAbs) led to increased production of IL-17 and expansion of IL-17^+^/CD4^+^ cells in lymph nodes. Surprisingly, percentage of Th17 cells infiltrating joints was lower after treatment and it was associated with reduced arthritis severity. Park et al. (2012) reported that IL-17 production in inflamed joint was promoted by TNF-like weak inducer of apoptosis. Contradictory results describing role of IL-22 and its interaction with IL-17 were observed in arthritis. Zhang et al. (2011) noticed that increase of Th17 cells was followed by proportional elevation of number of IL-22 producing cells and both parameters correlated with disease activity. On the other hand, recent publication of van Hamburg et al. (2013) reported that IL-17A/Th17 cell-mediated synovial inflammation was independent on IL-22 and Th22 cells.

The role of balance between pro-inflammatory Th17 cells and regulatory T lymphocytes in pathogenesis of autoimmune diseases has been repeatedly emphasized (Samson et al. 2012). Wang et al. (2012) and Niu et al. (2012) showed in their research the imbalance of Th17/Treg in the peripheral blood of patients with RA. Generation of Th17 cells is promoted by proinflammatory cytokines like IL-6 and IL-23 while the concentrations of TGF-β and the number of Treg cells are decreased in RA patients. According to Wang and co-workers (2012), these combined changes contributed to autoimmunity triggering and inflammation induction. Interestingly, Jiao et al. (2013) showed importance of myeloid-derived suppressor cells (MDSC) in controlling of Th17 cells, although MDSC number was correlated neirther with plasma level of IL-6 and IL-17 nor with the mRNA level of RORγt. The authors showed that MDSC negatively correlated with TNF level, what could prove that this population is in control of two independent mechanisms of RA pathogenesis related to increased secretion of TNF and IL-17. Moreover, some groups showed that proliferation of Th17 cells is activated by phosphorylation of signal transducer and activator of transcription 3 (STAT3) (Dong et al. 2014; Son et al. 2014). Inhibition of STAT3 leads to decrease of Th17 cell frequency and in consequence the number of Tregs is elevated.

IL-6 controls differentiation of Th17 cells via binding IL-6 to the IL-6 receptor’s α chain and to gp130, both of which mainly activate JAK1 and STAT3 (Tamiya et al. 2011). Surprisingly use of pyridone 6, a pan-JAK inhibitor, led to exacerbation as well as amelioration of autoimmune diseases (Nakagawa et al. 2011; Yoshida et al. 2012). The effect of inhibition of STAT3 activation and IL-6 signaling seems to be depending on other cells and cytokines involved in pathogenesis of particular disease. For example, IL-7 is another cytokine regulating Th cells differentiation via JAK and blockade of IL-7Rα potently inhibited autoimmune joint inflammation by reduction of Th17 activity (Hartgring et al. 2010). Activation of STAT3 can directly induce the production of IL-17 as well as IL-21, which are autocrined by differentiating Th17 cells and expand the differentiation of Th17 (Chen et al. 2007). Zhou et al. (2012) showed that fenobirate, and some other PPARα agonists, suppressed differentiation of Th17 cells in vitro via reducing of STAT3 activation and IL-21 secretion. RORγt was shown to be crucial factor for the differentiation of Th17 cells. Sustained activity of this factor is required for the function of differentiated Th17 lymphocytes. RORγt belongs to nuclear hormone receptor family, which makes it a good target for small molecule inhibitors, e.g. digoxin and ursolic acid (Huh et al. 2011; Huh and Littman 2012). Collagen-induced arthritis (CIA) is a popular animal model used for testing of Th17 targeting therapies. The crucial factor for development of arthritis in collagen-immunized mice is IL-23. However, recently published paper by Cornelissen et al. (2013) has shown that IL-23 is not a critical factor during the effector stage of CIA. Their results suggest that specific anti-IL-23p19 antibodies may not be beneficial as a therapeutic regime after onset of autoimmune arthritis for RA patients. On the other hand, T-cell-mediated arthritis relapses in patients with RA and might be controlled by neutralization of IL-23.

Th17-dependent pathways are controlled not only by cytokines, but also by neuromediators. Cholecystokinin octapeptide (CCK-8) and vasoactive intestinal peptide are the neuropeptides significantly suppressing the incidence and severity of CIA in mice. These factors also inhibit dendritic cell-mediated Th17 polarization (Deng et al. 2010; Li et al. 2011). Antagonist of D1-like receptor inhibits cartilage destruction in a human RA/SCID mouse chimera model and this effect is associated with decrease of dopamine-mediated IL-6 and IL-17 (Nakano et al. 2011).

Food products and diet could be used for control of Th17 activity. Kim et al. (2008) showed that rat fed with green tea significantly reduced the severity of arthritis compared with the water-fed controls and this effect was associated with decreased concentration of IL-17 and increased level of IL-10. Another plant derived agents—daphnetin and grape seed proanthocyanidin extract also reduced symptoms associated with CIA via decrease of IL-17 production (Cho et al. 2009; Tu et al. 2012). Plant extracts influencing function of Th17 cells and ameliorating severity of arthritis are not the only immunomodulatory substances taken from traditional medicine. Tanaka et al. (2012) showed in mouse model of arthritis that Brazilian propolis declined IL-17 expression measured by ELISA and real-time PCR methods. Additionally, Okamoto et al. (2012) noticed that propolis decreased differentiation of IL-17 producing cells and this effect was mediated via suppression of the IL-6-induced phosphorylation of STAT3.

The novel synthetic agents can also influence differentiation and activation of Th17 lymphocytes and attenuate rheumatoid inflammation in mice. In this group there is also all-trans retinoic acid (ATRA) (Kwok et al. 2012), which reduces IL-17 level and promotes activation of Treg cells in ATRA-treated mice. The next synthetic agent is tylophorine analog (NK-007) (Wen et al. 2012). It suppresses production of IL-6 and IL-17A in joints of mice with CIA. Similarly, polymerized-type I collagen causes decrease of IL-17A level and up-regulates the number of Tregs and Th1 cells (Furuzawa-Carballeda et al. 2012). Chen et al. (2010a) demonstrated in CIA mice that the suppression of PGE2 EP(4) receptor leads to decrease of cytokines production by Th1 and Th17 cells. Moreover, Moon et al. (2014) showed that rebamipide, a gastroprotective agent, is able to reduce inflammation in CIA mice by the recovery from an imbalance between Th17 and Treg cells.

Cyclosporine A (CsA) is an immunosuppressive drug used in transplantation medicine and treatment of autoimmune diseases. CsA reduces expression of IL-17 at protein and at mRNA levels in the cells isolated from the blood of patients with RA (Zhang et al. 2008). Similar effect was described in patients with Vogt-Koyanagi-Harada syndrome treated with CsA combined with corticosteroids (Liu et al. 2009). Contrary to these results, Abadja et al. (2009) reported that CsA did not inhibit IL-17 expression in cultured human peripheral blood mononuclear cells. Also, Lemaître et al. (2013) showed that CsA failed to inhibit IL-17 production in mice after heterotopic trachea transplantation.

### Juvenile Idiopathic Arthritis

Juvenile idiopathic arthritis (JIA) is a heterogeneous and multifactorial autoimmune pediatric disease characterized by chronic joint inflammation with onset age younger than 16 years. It is the most common chronic rheumatic disease in childhood and an important cause of disability. JIA has different subtypes that are defined based on the number of inflamed joints during the first 6 months of disease and the extra-articular involvement. In JIA patients, similarly to adult patients with RA, joint microenvironment promotes Th17 differentiation. Cosmi et al. (2011) noticed increased number of Th17/Th1 cells described as producing both IFN-γ and IL-17A, but not IL-17A alone in children with oligoartricular onset of JIA. They also reported that CD4^+^/CD161^+^ cells producing only IL-17A can easily shift into cells producing IFN-γ and IL-17A or IFN-γ alone. Synovial Th17 cells produce also IL-22 and IFN-γ, but not IL-4. The number of T cells producing IL-17 is higher in the joints of patients with the more severe phenotype of disease (Nistala et al. 2008). Expression of CCR6 and response to CCL20 were also confirmed in JIA patients, so it is in accordance with described mechanisms of Th17 cell migration in RA patients. In JIA patients it was also confirmed that reciprocal relations between Th17 and Treg cells are crucial in development of chronic inflammation. Systemic subtype of JIA is associated with anomalies in the innate immune system leading to the loss of control of alternative secretory pathway in phagocytes. Omoyinmi et al. (2012) noticed increased percentage of IL-17^+^CD3^+^ both CD4^+^ and CD4^−^ comparing to healthy age matched controls, but not in comparison to oligoarticular JIA.

### Sjögren Syndrome, Systemic Sclerosis, Ankylosing Spondylitis

The presence of Th17 lymphocytes and IL-17 at the protein and mRNA levels were confirmed in inflamed salivary glands and peripheral blood of patients with Sjögren syndrome (Ciccia et al. 2012; Katsifis et al. 2009; Maehara et al. 2012). Increased activity of Th17 cells was associated with low number of T regulatory cells and higher concentration of IL-21, IL-22, IL-23 (Ciccia et al. 2012; Kang et al. 2011). Alunno et al. (2013) noticed that CD4^−^CD8^−^ T cells producing IL-17 from patients with Sjögren syndrome were in vitro resistant to corticosteroids. Additionally influence of IL-17R on human salivary cells lines was confirmed and IL-17 stimulated salivary gland cells to secrete pro-inflammatory IL-6 and IL-8 (Sakai et al. 2008).

IL-17A-producing T cells are characteristically increased in peripheral blood of patients with systemic sclerosis (SSc) and they are characterized by expression of chemokine receptor CCR6 responsible for with skin- and lung-homing capabilities (Radstake et al. 2009; Truchetet et al. 2011). Results describing plasma level of IL-17A remain ambiguous. Murata et al. (2008) described elevated concentration of IL-17A in the blood of SSc patients. On the contrary Radstake et al. (2009) did not find significant differences in IL-17A concentration between SSc individuals and healthy controls. The increased percentage of circulating Th17 cells is rather independent on type or stage of disease. Additionally, frequency of Th17 cells in bronchoalveolar lavage fluid of patients with SSc was associated with lungs’ involvement (Fenoglio et al. 2011; Meloni et al. 2009). The presence of Th17 in blood and affected tissues seems to be crucial for SSc development. Surprisingly, the recent study of Truchetet et al. (2013) has shown that the presence of IL-17A^+^ cells correlates inversely with the extent of skin fibrosis. Th17 cells may counterbalance fibrosis via induction of extracellular matrix degrading enzymes, inhibition of collagen synthesis, and myofibroblast differentiation, while favoring a pro-inflammatory microenvironment.

The number of Th17 cells and concentration of IL-17 and IL-23 are increased in the peripheral blood of patients with ankylosing spondylitis (Mei et al. 2011; Shen et al. 2009; Zhang et al. 2012). IL-17 producing cells expressed phenotype of memory cells CD4^+^CD45RO^+^ and were positive for CCR6 (Shen et al. 2009). Percentage of Th17 cells and concentrations of IL-17 and IL-23 positively correlated with disease activity and were increased in patients not responding to anti-TNF therapy (Xueyi et al. 2013).

## Organ-Specific Autoimmune Diseases

The autoimmune organ-specific diseases are mediated by specific immune response directed against antigens characteristic for particular tissues. The role of Th1 and Th2 in this type of disorders was quite well established. Th17 seems to be a new player in organ specific autoimmunity.

### Type 1 Diabetes

Type 1 diabetes (T1D) is an autoimmune disease in which insulin-producing β cells in the pancreatic islets are destroyed by cytotoxic T lymphocytes, resulting in deficiency of insulin and chronic hyperglycemia requiring lifelong dependence on insulin supplementation. Upregulation of Th17 immunity in peripheral blood and lymph nodes of patients with T1D was observed (Ferraro et al. 2011; Honkanen et al. 2010). Dysregulation of Th17 and Treg interaction was also confirmed. More interestingly, Marwaha et al. (2010) showed that CD45RAneg/CD25mid/FOXP3low lymphocytes lost regulatory abilities and secreted IL-17, what made them pro-inflammatory cells.

### Autoimmune Thyroid Disorders

The most common autoimmune thyroid diseases (AITD) are Graves’ disease in young adults and Hashimoto’s disease in children and young women. The proportion of peripheral Th17 cells in patients with AITD was higher than in control subjects and it was dependent on disease activity and severity (Figueroa-Vega et al. 2010; Kim et al. 2012; Nanba et al. 2009). However, Zheng et al. (2013) reported increased expression of IL-17 mRNA also in euthyroid patients with Graves’ disease and it was upregulated after stimulation with IL-23. Bossowski et al. (2012) examined a group of children with AITD and they found increased percentage of CD4^+^IL-17^+^ cells in children with untreated Hashimoto’s disease, but not in children with Graves’ disease. Additionally, percentage of CD4^+^IL-17^+^ cells positively correlated with titers of anti-thyroid peroxidase immunoglobulins. Shi et al. (2010) suggested that Th17 cells can play a central role in pathogenesis of Hashimoto’s disease rather than Th1 cells. Thesr conclusions were based on confirmed higher expression of IL-17 mRNA than IFN-γ mRNA in peripheral blood cells. Unfortunately, authors did not include any data about duration of disease what in combination with age of patients ranged 23–60 leading to conclusions that patients were rather not in active phase of lymphocytic thyroiditis. This could explain low activity of Th1 response and relative predominance of IL-17 producing cells.

### Multiple Sclerosis, Myasthenia Gravis

Multiple sclerosis (MS) is a chronic inflammatory disease with destruction of myelin shift in central nervous system. The blood–brain barrier (BBB) disruption is an early and main event in MS development. Autoreactive Th17 cells can disrupt tight junction proteins in the central nervous system endothelial cells and migrate through the BBB and this effect is mediated via IL-17 and IL-22 (Kebir et al. 2007). Accumulation of Th17 cells was found in cerebrospinal fluid of patients with MS relapse. The number of Th17 was higher in affected individuals than in patients in remission or patients with other central nervous system pathologies (Brucklacher-Waldert et al. 2009; Schirmer et al. 2013). These cells were characterized by high expression of activation markers and molecules involved in T cell adhesion to endothelium. Christensen et al. (2013) reported that percentage of circulating Th17 cells, identified as CD4^+^IL-23R^+^ cells, was increased in patients with more severe type of MS. These results stay in accordance with previous results describing increased percentage of CD3^+^CD4^+^IL-17^+^ and CD3^+^CD8^+^IL-17^+^ cells in patients with MS (Wang et al. 2011).

Several studies were focused on changes in IL-17 serum concentration during therapy with IFN-β. All studies confirmed decrease of IL-17 induced by IFN-β, although in studies analyzing response to therapy this effect was described only in patients responding to therapy (Esendagli et al. 2013; Kürtüncü et al. 2012; Kvarnström et al. 2013). Additionally, patients with high serum IL-17A levels did not respond well to IFN-β therapy (Balasa et al. 2013).

Multiple sclerosis is still incurable disease, where blockade of Th17 cells could be useful in treatment, so a set of studies utilizing experimental autoimmune encephalomyelitis (EAE) as animal model of demyelinating disorders was conducted. Nakano et al. (2008) showed that use of a dopamine D1-like-receptor antagonist inhibited dendritic cell-mediated Th17 differentiation and prevented development of EAE as well as had therapeutic effect in mice model.

Similarly to experiments focused on arthritis also in autoimmune brain disorders we could control activity of Th17 cells with some food ingredients. Active ingredient of green tea epigallocatechin-3-gallate also reduced clinical symptoms of EAE in dose-dependent manner and this result was due to reduced production of Th17 associated cytokines and decreased percentage of CD4^+^ cells producing IL-17 (Wang et al. 2012). Similar results were observed in mice treated with ghrelin, anatabine, eriocalyxin, salmon cartilage proteoglycans (Lu et al. 2013; Paris et al. 2013; Sashinami et al. 2012; Souza-Moreira et al. 2013).

Roche et al. (2011) have shown that IL-17 concentration was higher in myasthenia gravis patients compared with controls and it positively correlated with anti-acetylcholinesterase receptor antibody titers.

### Inflammatory Bowel Diseases

Crohn’s disease and ulcerative colitis are chronic IBDs thought to be caused by an abnormal immune response directed against normal constituents of the intestinal flora. IL-17 expression and percentages of Th17 and Th1/Th17 in the inflamed mucosa and serum was increased in IBD patients (Fujino et al. 2003; Nielsen et al. 2003; Rovedatti et al. 2009). Surprisingly, Rovedatti et al. (2009) reported that production of IFN-γ in inflamed mucosa of patients with ulcerative colitis and Crohn’s disease was equivalent to normal mucosa and that TGF-β inhibited its production, but did not downregulate production of IL-17. On the other hand, Kobayashi et al. (2008) reported that the transcripts for Th17-related cytokines were more abundant in ulcerative colitis than in Crohn’s disease when the two diseases were compared and IL-23 enhanced production of IL-17 in lamina propria of patients with ulcerative colitis, while IFN-γ in patients with Crohn’s disease. Based on these data, authors suggested that ulcerative colitis may actually be classified as a Th17 disease, while Crohn’s disease remains a Th1 disease. Similar results were reported for peripheral blood cells (Raza and Shata 2012; Veny et al. 2010). Although, other researchers confirmed activation of Th17 pathway in intestinal mucosa and mesenteric lymph nodes in patients with Crohn’s disease (Hovhannisyan et al. 2011; Hölttä et al. 2008; Sakuraba et al. 2009).

Animal models confirm that Th17 cells and cytokines associated with this population play crucial role in development of IBD and show great potential as targets for therapy of autoimmune bowel’s diseases. Fina et al. (2008) studied the potential influence of IL-21 on Th17 subpopulation and development of gut inflammation. Researchers demonstrated that IL-21-deficient mice were protected against colitis and were unable to increase the number of Th17 cells. Moreover, neutralization of IL-21 in wild-type mice with the use of anti-IL-21 antibody decreased the level of IL-17. However, there are some papers displaying differences between suppression of IL-17A and IL-17F. The development of acute mucosal inflammation was impossible in trinitrobenzene sulfonic acid induced colitis in mice with IL-17RA deficiency (Wallace et al. 2014). However, in a dextran sodium sulfate model of colitis IL-17F deficiency suppressed mucosal inflammation, but the lack of IL-17A stimulation caused exacerbation of IBD. Indeed, IL-17A may be protection factor against the development of IBD, and IL-17F may contribute to mucosal inflammation. However, this concern should be deeply investigated, because there are also some results suggesting that IL-17A exacerbates IBD. Zhang et al. (2014) showed, that heme oxygenase-1 (HO-1) decreased the number of Th17 cells and causes an increase of Treg population in murine model of acute experimental colitis. HO-1 switched the naïve T cells to Tregs and reduced the expression of RORγt and level of IL-17A. Therefore, HO-1 might provide a novel therapeutic target in IBD.

### Psoriasis

Psoriasis is a chronic inflammatory skin disease associated with complex skin inflammatory process resulting in keratinocyte hyperplasia. Increased frequency of Th17 cells was confirmed in skin lesions and peripheral blood of patients with psoriasis (Kagami et al. 2010; Lowes et al. 2008). Arican et al. (2005) showed that serum concentration of IL-17 did not differ from healthy donor, but proved significant correlation with disease activity. These results are not corresponding with the Coimbra et al. (2010) study reporting increased IL-17 concentration in psoriatic patients when compared to healthy individuals. Additionally, the level of IL-17 lowered during UV therapy and it was followed by improvement of clinical symptoms. Treatment with soluble TNF receptor exacerbates murine psoriasis-like disease and symptoms are related to increased expression of the Th17 associated cytokines such as IL-1β, IL-6, IL-17, IL-21, IL-22, and reduction of Treg cells (Ma et al. 2010).

The systemic and local activity of IL-17 and Th17 seems to be important part of development of autoimmune reaction. The ability to induce pro-inflammatory function of the cells outside immune systems, e.g. synovial cells, is one of the factors leading to chronic inflammation. Disturbances in balance between Th17 lymphocytes and regulatory cells (Treg and myeloid derived suppressor cells) are described in the majority of autoimmune diseases. Correlation between disease activity and concentration of IL-17 or percentage of Th17 in the peripheral blood or other body fluids could be additional laboratory parameter used in clinical prognosis and monitoring of clinical course of the disease.

## Th17 Lymphocytes and Their Pathways as Target for Therapy: Clinical Trials

**Fig. 1:**
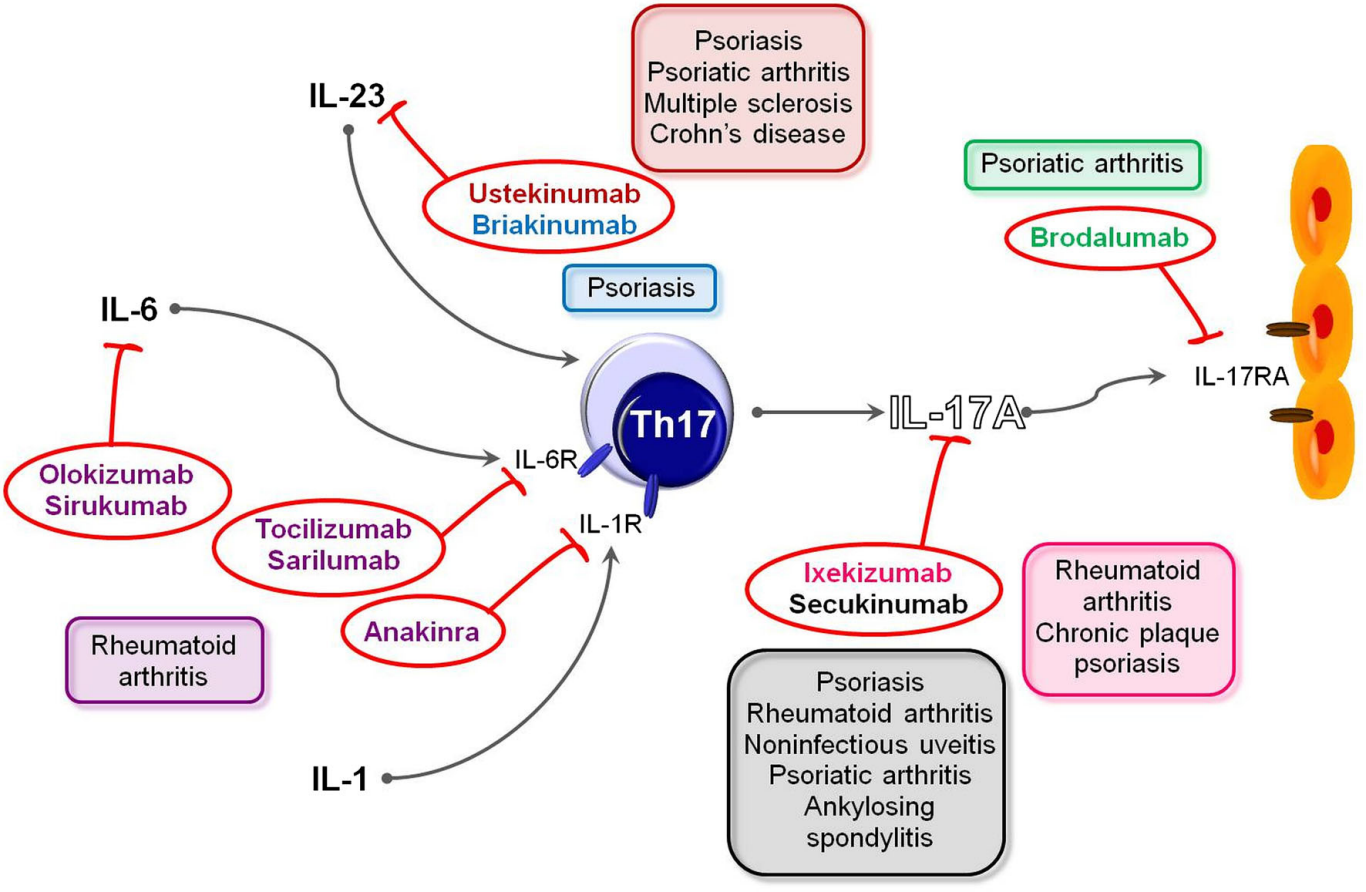
Drugs targeting Th17 pathways and their use in human diseases

Although the role of Th17 lymphocytes in etiology and pathology of human diseases is quite different and multifaceted, both in vitro and in vivo studies have clearly demonstrated the pivotal role of IL-17 in the pathogenesis of various diseases, making IL-17 a perfect target for therapeutic procedures. On the basis of pre-clinical studies, immunologists predicted that blocking of IL-17/IL-17R or cytokines that promote Th17 induction or activation (IL-1, IL-6 or IL-23) could be beneficial mainly in patients with chronic inflammatory diseases. The mAbs targeting these pathways and potential use in autoimmune diseases is shown in Fig. 1. On the other hand, questions and skepticism surround the usefulness of animal models as direct predictors of potential therapeutics for IL-17-based drugs, so in consequence the results of human clinical trials targeting this pathway have been fervently awaited.

In patients with psoriasis or IBDs, treatment with different TNF inhibitors enhances Th17 activity in blood, whereas in the inflamed tissues of responding patients, it induces a potent shut down of Th17-Th1 cytokines (Bosè et al. 2011). Sugita et al. (2012) showed that anti-TNF chimeric antibody (Infliximab) inhibited Th17 differentiation in uveitis patients with Behçet’s disease. Downregulation of Th17 number and functions seems to be important to achieve therapeutic effect of anti-TNF treatment in patients with RA. Studies based on human peripheral blood cells showed that number of IL-17 producing lymphocytes and concentrations of cytokines associated with Th17 development and functions are decreased in patients who responded to treatment, while in non-responding patients these levels and functions were elevated (Alzabin et al. 2012; Chen et al. 2011; Zivojinovic et al. 2012). Additionally, the study of van Hamburg et al. (2012) suggested that 1,25(OH)_2_D_3_ could be crucial for suppression of Th17 cells by anti-TNF treatment. Also combination of TNF inhibitors and methotrexate is more efficient in downregulation of Th17 response than soluble TNF receptor alone (Lina et al. 2011).

The majority of conducted clinical trials were focused on psoriasis, what led to approval of ustekinumab (fully human IgG1 mAb against p40 subunits of IL-12/IL-23) for treatment of moderate to severe plaque psoriasis (Patel et al. 2013; Wofford and Menter 2014). Moreover, the usefulness of ustekinumab was also evaluated in other immune-mediated diseases such as psoriatic arthritis, MS and Crohn’s disease. Results of these trials are summarized in Table 1. In Crohn’s disease the effectiveness of ustekinumab was achieved most notably in patients who had received previously biological therapy. Other researchers showed that the clinical response was better in ustekinumab group than in placebo series in patients resistant to TNF inhibitors (Toussirot et al. 2013; Tuskey and Behm 2014). The study comparing ustekinumab with etanercpet showed ustekinumab’s superiority to high-dose etanercept in managing of moderate-to-severe psoriasis (Griffiths et al. 2010). However, a phase 2 clinical trials investigating efficacy of ustekinumab in MS displayed its inefficiency in reducing the number of enhancing lesions (Ghosh 2012; Scherl et al. 2010). Despite encouraging results showing clinical benefits of IL-17 and IL-23 inhibition, recently published meta-analyses have underlined higher number of major adverse effects in comparison to placebo group and suggested that patients shall be monitored for these potential safety signals (Langley et al. 2013; Spuls and Hooft 2012; Tzellos et al. 2013). Clinical trials focused on other mAbs are summarized in Table 2.

**Table 1:** Clinical studies of ustekinumab in psoriasis, psoriatic arthritis, Crohn’s disease, and multiple sclerosis

Study	Immune-mediated disease	Study design	Results
Krueger et al. (2007)	Psoriasis	Phase II	Ustekinumab: 52–81 % Placebo: 2 %
Leonardi et al. (2008) (PHOENIX 1)	Psoriasis	Phase III	Ustekinumab: 67 % Placebo: 3 %
Papp et al. (2008) (PHOENIX 2)	Psoriasis	Phase III	Ustekinumab: 67–76 % Placebo: 4 %
Griffiths et al. (2010) (ACCEPT)	Psoriasis	Phase III head to head comparative study	Ustekinumab: 68–74 % Etanercept: 57 %
Gottlieb et al. (2009)	Psoriatic arthritis	Phase II	Group 1 (ustekinumab then placebo) versus group 2 (placebo then ustekinumab) 42 vs 14 %
Sandborn et al. (2008)	Crohn’s disease	Phase II	Ustekinumab: 49 % Placebo: 40 %
Sandborn et al. (2012)	Crohn’s disease	Phase III	Ustekinumab: 36–39 % Placebo: 23.5 %
Segal et al. (2008)	Multiple sclerosis	Phase II	No difference between any ustekinumab groups vs placebo

Results are presented as the percentage of patients who reached the primary endpoint [a 75 % reduction in the Psoriasis Area and Severity Index score (PASI75)] (Toussirot et al. 2013)

**Table 2:** Clinical studies for using Th17 pathways as a target for therapy

Agent	Target	Trial phase	Disease	References
Ustekinumab	IL-12/IL-23p40	I	Psoriasis	Kauffman et al. (2004)
		I	Psoriasis	Gottlieb et al. (2007)
		II	Psoriasis	Krueger et al. (2007)
		III	Psoriasis	Griffiths et al. (2010)
		III	Psoriasis	Papp et al. (2008)
		III	Psoriasis	Leonardi et al. (2008)
		I	Multiple sclerosis	Kasper et al. (2006)
		II	Multiple sclerosis	Segal et al. (2008)
Briakinumab	IL-12/IL-23p40	III	Psoriasis	Eggleton et al. (2011)
		III	Psoriasis	Kauffman et al. (2004)
Brodalumab	IL-17RA	II	Psoriasis	Gottlieb et al. (2007)
Ixekizumab	IL-17A and IL-17A-IL-17F hetoridmers	II	Psoriasis	Krueger et al. (2007)
		I	Rheumatoid arthritis	Papp et al. (2008)
Secukinumab	IL-17A	II	Psoriasis	Leonardi et al. (2008)
		II	Psoriasis	Kasper et al. (2006)
		II	Psoriatic arthritis	Chioato et al. (2012)
		II	Rheumatoid arthritis	Krueger et al. (2007)
		II	Psoriasis/rheumatoid arthritis/uveitis	Leonardi et al. (2008)
		II	Crohn’s disease	Hueber et al. (2012)
		III	Noninfectious uveitis	Dick et al. (2013)
		I	Vaccinated healthy adults	Chioato et al. (2012)
Tocilizumab	IL-6R	Approved as therapy	Rheumatoid arthritis	Hernández et al. (2013)
Anakinra	IL-1R	Approve as therapy	Rheumatoid arthritis	Cavagna and Taylor (2014)
Olokizumab	IL-6	II	Rheumatoid arthritis	Tanaka and Mola (2014)
Sarilumab	IL-6Rα	II	Rheumatoid arthritis	Huizinga et al. (2014)
Sirukumab	IL-6	II	Rheumatoid arthritis	Smolen et al. (2014)

IL-12/23p40 pathway is quite popular in therapy for autoimmune diseases. There are some more pharmaceutical substances using this pathway to inhibit Th17 cells. Briakinumab is one among them. Reich et al. (2011) showed in third phase clinical trial that it can be more effective in psoriasis than methotrexate. It also caused more adverse events; however the difference was statistically insignificant.

Brodalumab has been proposed for therapy of psoriatic arthritis. It is a human monoclonal anti-IL-17A receptor antibody. The results of second phase clinical trial displayed that brodalumab improved symptoms in patients with psoriatic arthritis compared to placebo group (Mease et al. 2014). Ixekizumab (LY2439821) has been evaluated in second phase clinical study of RA (Genovese et al. 2014) and chronic plaque psoriasis (Tham et al. 2014). This humanized mAb selectively binds to IL-17A and neutralizes this pro-inflammatory cytokine. Furthermore, ixekizumab was proved to be effective also in difficult-to-treat areas, e.g. scalp and nails (Spuls and Hooft 2012).

Secukinumab (AIN457, a recombinant, highly selective, fully human IgG1 k mAb against IL-17A) was shown to improve clinical symptoms in patients with psoriasis, RA and noninfectious uveitis. Moreover, it enabled reduction of symptoms in psoriatic arthritis and ankylosing spondylitis. In the second phase clinical trial was shown, that in both cohorts: psoriasis/psoriatic arthritis and RA, patients treated with secukinumab have shown more effective therapeutic response in comparison to placebo group. Studies displayed improvement of rates such as psoriasis area and severity index scores in psoriasis patients and rate of American College of Rheumatology 20 score in RA. Rates of adverse events (AEs) and serious AEs were comparable between secukinumab and placebo groups in psoriasis but in rheumatoid arthritis AEs rate was higher in secukinumab cohort (Patel et al. 2013). Dick et al. (2013) described three clinical trials in third phase which evaluated the effectiveness of secukinumab in treatment of noninfectious uveitis. They did not notice any statistical significance between secukinumab and placebo groups. Hueber et al. (2012) published unexpected results describing ineffectiveness of secukinumab in controlling severe Crohn’s disease. Additionally they revealed higher rates of adverse events in mAb treated group comparing to placebo series.

An anti-IL6 receptor antibody (tocilizumab) and an IL-1 receptor antagonist (anakinra), which were approved for therapy of human autoimmune diseases, also target development and function of Th17 cells (Samson et al. 2012). ADACTA study displayed that tocilizumab is more effective in RA monotherapy than adalimumab (TNF antagonist) while AMBITION study revealed that tocilizumab may be considered as the first biological drug more effective than methotrexate (Hernández et al. 2013). Samson et al. (2012) demonstrated that tocilizumab may correct of imbalance between Th17 and Treg cells in patients with RA. It is worth emphasizing that it was well tolerated after subcutaneous or intravenous administration (Burmester et al. 2014; Kivitz et al. 2014). Anakinra is the first IL-1R antagonist accepted in therapy of RA and it binds to both IL-1α and IL-1β (Cavagna and Taylor 2014).

Tofacitinib (CP-690,550) is a novel JAK/STAT inhibitor directly suppressing the production of IL-17 and IFN-γ and its effect is associated with decreased concentration of IL-6 (Ghoreschi et al. 2011; Maeshima et al. 2012). This Janus kinase inhibitor was proved to enhance clinical status of patients with RA in randomized, double-blind, placebo-controlled phase 2a trial (Coombs et al. 2010).

Olokizumab, sarilumab, and sirukumab are some other drugs using blocade of IL-6 pathway in their interactions. The first is a humanized anti-IL-6 antibody, which in 2b phase clinical trial was proved to be as effective as tocilizumab in therapy of RA (Tanaka and Mola 2014). Sarilumab, a human mAb directed against IL-6Rα, can also improve symptoms of RA, which was showed in the second phase clinical trials (Huizinga et al. 2014). However, it was shown to be ineffective in ankylosing spondylitis therapy (Sieper et al. 2014). The last drug—sirukumab (anti-IL-6 mAb)—was also assessed in the second phase clinical trials in RA. In results the researchers noted an improvement of clinical status of patients treated with sirukumab when compared with placebo group (Smolen et al. 2014).

Furthermore, Eggleton et al. (2011) presented that a greater proportion of Th17 cells isolated from the peripheral blood of RA patients expressed CD20 in contrast to healthy individuals. These cells may represent an additional target for popular anti-CD20 therapies.

## Conclusions

Th17 cell population has given immunologists and clinicians some new possibilities. Researchers exploring molecular pathways connected with Th17 cells try to find more effective therapies of autoimmune disease. Whether biological drugs based on inhibition of Th17 dependent pathways will emerge as front-line therapies for autoimmune and chronic inflammatory diseases is still unclear. The data from phase three clinical trials are moderately encouraging but thus far not overwhelming when considered alongside with other existent or developing therapeutics.

## References

[CR1] Abadja F, Videcoq C, Alamartine E (2009). Differential effect of cyclosporine and mycophenolic acid on the human regulatory T cells and TH-17 cells balance. Transplant Proc.

[CR2] Adami S, Cavani A, Rossi F (2014). The role of interleukin-17A in psoriatic disease. BioDrugs.

[CR3] Alunno A, Bistoni O, Bartoloni E (2013). IL-17-producing CD4-CD8- T cells are expanded in the peripheral blood, infiltrate salivary glands and are resistant to corticosteroids in patients with primary Sjogren’s syndrome. Ann Rheum Dis.

[CR4] Alzabin S, Abraham SM, Taher TE (2012). Incomplete response of inflammatory arthritis to TNFα blockade is associated with the Th17 pathway. Ann Rheum Dis.

[CR5] Arican O, Aral M, Sasmaz S (2005). Serum levels of TNF-alpha, IFN-gamma, IL-6, IL-8, IL-12, IL-17, and IL-18 in patients with active psoriasis and correlation with disease severity. Mediators Inflamm.

[CR6] Arroyo-Villa I, Bautista-Caro MB, Balsa A (2012). Frequency of Th17 CD4+ T cells in early rheumatoid arthritis: a marker of anti-CCP seropositivity. PLoS One.

[CR7] Balasa R, Bajko Z, Hujtanu A (2013). Serum levels of IL-17A in patients with relapsing-remitting multiple sclerosis treated with interferon-β. Mult Scler.

[CR8] Bosè F, Raeli L, Garutti C (2011). Dual role of anti-TNF therapy: enhancement of TCR-mediated T cell activation in peripheral blood and inhibition of inflammation in target tissues. Clin Immunol.

[CR9] Bossowski A, Moniuszko M, Idźkowska E (2012). Evaluation of CD4+CD161+CD196+ and CD4+IL-17+ Th17 cells in the peripheral blood of young patients with Hashimoto’s thyroiditis and Graves’ disease. Pediatr Endocrinol Diabetes Metab.

[CR10] Brucklacher-Waldert V, Stuerner K, Kolster M (2009). Phenotypical and functional characterization of T helper 17 cells in multiple sclerosis. Brain.

[CR11] Burmester GR, Rubbert-Roth A, Cantagrel A (2014). A randomised, double-blind, parallel-group study of the safety and efficacy of subcutaneous tocilizumab versus intravenous tocilizumab in combination with traditional disease-modifying antirheumatic drugs in patients with moderate to severe rheumatoid arthritis (SUMMACTA study). Ann Rheum Dis.

[CR12] Cai Z, Zhang W, Yang F (2012). Immunosuppressive exosomes from TGF-β1 gene-modified dendritic cells attenuate Th17-mediated inflammatory autoimmune disease by inducing regulatory T cells. Cell Res.

[CR13] Cavagna L, Taylor WJ (2014). The emerging role of biotechnological drugs in the treatment of gout. Biomed Res Int.

[CR14] Chabaud M, Durand JM, Buchs N (1999). Human interleukin-17: a T cell-derived proinflammatory cytokine produced by the rheumatoid synovium. Arthritis Rheum.

[CR15] Chen Z, Laurence A, O’Shea JJ (2007). Signal transduction pathways and transcriptional regulation in the control of Th17 differentiation. Semin Immunol.

[CR16] Chen Q, Muramoto K, Masaaki N (2010). A novel antagonist of the prostaglandin E(2) EP(4) receptor inhibits Th1 differentiation and Th17 expansion and is orally active in arthritis models. Br J Pharmacol.

[CR17] Chen XQ, Yu YC, Deng HH (2010). Plasma IL-17A is increased in new-onset SLE patients and associated with disease activity. J Clin Immunol.

[CR18] Chen DY, Chen YM, Chen HH (2011). Increasing levels of circulating Th17 cells and interleukin-17 in rheumatoid arthritis patients with an inadequate response to anti-TNF-α therapy. Arthritis Res Ther.

[CR19] Chioato A, Noseda E, Stevens M (2012). Treatment with the interleukin-17A-blocking antibody secukinumab does not interfere with the efficacy of influenza and meningococcal vaccinations in healthy subjects: results of an open-label, parallel-group, randomized single-center study. Clin Vaccine Immunol.

[CR20] Cho ML, Heo YJ, Park MK (2009). Grape seed proanthocyanidin extract (GSPE) attenuates collagen-induced arthritis. Immunol Lett.

[CR21] Christensen J, Börnsen L, Ratzer R (2013). Systemic inflammation in progressive multiple sclerosis involves follicular T-helper, th17- and activated B-cells and correlates with progression. PLoS One.

[CR22] Ciccia F, Guggino G, Rizzo A (2012). Potential involvement of IL-22 and IL-22-producing cells in the inflamed salivary glands of patients with Sjogren’s syndrome. Ann Rheum Dis.

[CR23] Coimbra S, Oliveira H, Reis F (2010). Interleukin (IL)-22, IL-17, IL-23, IL-8, vascular endothelial growth factor and tumour necrosis factor-α levels in patients with psoriasis before, during and after psoralen-ultraviolet A and narrowband ultraviolet B therapy. Br J Dermatol.

[CR24] Coombs JH, Bloom BJ, Breedveld FC (2010). Improved pain, physical functioning and health status in patients with rheumatoid arthritis treated with CP-690,550, an orally active Janus kinase (JAK) inhibitor: results from a randomised, double-blind, placebo-controlled trial. Ann Rheum Dis.

[CR25] Cornelissen F, Asmawidjaja PS, Mus AM (2013). IL-23 dependent and independent stages of experimental arthritis: no clinical effect of therapeutic IL-23p19 inhibition inc-induced arthritis. PLoS One.

[CR26] Cosmi L, Cimaz R, Maggi L (2011). Evidence of the transient nature of the Th17 phenotype of CD4+CD161+ T cells in the synovial fluid of patients with juvenile idiopathic arthritis. Arthritis Rheum.

[CR27] Crispín JC, Oukka M, Bayliss G (2008). Expanded double negative T cells in patients with systemic lupus erythematosus produce IL-17 and infiltrate the kidneys. J Immunol.

[CR28] Deng S, Xi Y, Wang H (2010). Regulatory effect of vasoactive intestinal peptide on the balance of Treg and Th17 in collagen-induced arthritis. Cell Immunol.

[CR29] Dick AD, Tugal-Tutkun I, Foster S (2013). Secukinumab in the treatment of noninfectious uveitis: results of three randomized, controlled clinical trials. Ophthalmology.

[CR30] Dong L, Wang X, Tan J (2014). Decreased expression of microRNA-21 correlates with the imbalance of Th17 and Treg cells in patients with rheumatoid arthritis. J Cell Mol Med.

[CR31] Doreau A, Belot A, Bastid J (2009). Interleukin 17 acts in synergy with B cell-activating factor to influence B cell biology and the pathophysiology of systemic lupus erythematosus. Nat Immunol.

[CR32] Du J, Li Z, Shi J (2014). Associations between serum interleukin-23 levels and clinical characteristics in patients with systemic lupus erythematosus. J Int Med Res.

[CR33] Eggleton P, Bremer E, Tarr JM (2011). Frequency of Th17 CD20+ cells in the peripheral blood of rheumatoid arthritis patients is higher compared to healthy subjects. Arthritis Res Ther.

[CR34] Esendagli G, Kurne AT, Sayat G (2013). Evaluation of Th17-related cytokines and receptors in multiple sclerosis patients under interferon beta-1 therapy. J Neuroimmunol.

[CR35] Fenoglio D, Battaglia F, Parodi A (2011). Alteration of Th17 and Treg cell subpopulations co-exist in patients affected with systemic sclerosis. Clin Immunol.

[CR36] Ferraro A, Socci C, Stabilini A (2011). Expansion of Th17 cells and functional defects in T regulatory cells are key features of the pancreatic lymph nodes in patients with type 1 diabetes. Diabetes.

[CR37] Figueroa-Vega N, Alfonso-Pérez M, Benedicto I (2010). Increased circulating pro-inflammatory cytokines and Th17 lymphocytes in Hashimoto’s thyroiditis. J Clin Endocrinol Metab.

[CR38] Fina D, Sarra M, Fantini MC (2008). Regulation of gut inflammation and th17 cell response by interleukin-21. Gastroenterology.

[CR39] Fujino S, Andoh A, Bamba S (2003). Increased expression of interleukin 17 in inflammatory bowel disease. Gut.

[CR40] Furuzawa-Carballeda J, Macip-Rodríguez P, Galindo-Feria AS (2012). Polymerized-type I collagen induces upregulation of Foxp3-expressing CD4 regulatory T cells and downregulation of IL-17-producing CD4^+^ T cells (Th17) cells in collagen-induced arthritis. Clin Dev Immunol.

[CR41] Genovese MC, Greenwald M, Cho CS (2014). A phase II randomized study of subcutaneous ixekizumab, an anti-interleukin-17 monoclonal antibody, in rheumatoid arthritis patients who were naive to biologic agents or had an inadequate response to tumor necrosis factor inhibitors. Arthritis Rheumatol.

[CR42] Ghilardi N, Ouyang W (2007). Targeting the development and effector functions of TH17 cells. Semin Immunol.

[CR43] Ghoreschi K, Jesson MI, Li X (2011). Modulation of innate and adaptive immune responses by tofacitinib (CP-690,550). J Immunol.

[CR44] Ghosh S (2012). Biologic therapies: lessons from multiple sclerosis. Dig Dis.

[CR45] Gottlieb AB, Cooper KD, McCormick TS (2007). A phase 1, double-blind, placebo-controlled study evaluating single subcutaneous administrations of a human interleukin-12/23 monoclonal antibody in subjects with plaque psoriasis. Curr Med Res Opin.

[CR46] Gottlieb A, Menter A, Mendelsohn A (2009). Ustekinumab, a human interleukin-12/23 monoclonal antibody for psoriatic arthritis: randomised, double-blind, placebo-controlled, cross-over trial. Lancet.

[CR47] Griffiths CE, Strober BE, van de Kerkhof P (2010). Comparison of ustekinumab and etanercept for moderate-to-severe psoriasis. N Engl J Med.

[CR48] Hartgring SA, Willis CR, Alcorn D (2010). Blockade of the interleukin-7 receptor inhibits collagen-induced arthritis and is associated with reduction of T cell activity and proinflammatory mediators. Arthritis Rheum.

[CR49] Henriques A, Inês L, Couto M (2010). Frequency and functional activity of Th17, Tc17 and other T-cell subsets in systemic lupus erythematosus. Cell Immunol.

[CR50] Hernández MV, Vidal S, Sanmarti R (2013). Analysis of the mechanism of action of biological therapies in monotherapy in patients with rheumatoid arthritis: beyond the ADACTA Study. Adv Pharmacoepidemiol Drug Saf.

[CR51] Hirota K, Yoshitomi H, Hashimoto M (2007). Preferential recruitment of CCR6-expressing Th17 cells to inflamed joints via CCL20 in rheumatoid arthritis and its animal model. J Exp Med.

[CR52] Hölttä V, Klemetti P, Sipponen T (2008). IL-23/IL-17 immunity as a hallmark of Crohn’s disease. Inflamm Bowel Dis.

[CR53] Honkanen J, Nieminen JK, Gao R (2010). IL-17 immunity in human type 1 diabetes. J Immunol.

[CR54] Hot A, Miossec P (2011). Effects of interleukin (IL)-17A and IL-17F in human rheumatoid arthritis synoviocytes. Ann Rheum Dis.

[CR55] Hovhannisyan Z, Treatman J, Littman DR (2011). Characterization of interleukin-17-producing regulatory T cells in inflamed intestinal mucosa from patients with inflammatory bowel diseases. Gastroenterology.

[CR56] Hu Y, Shen F, Crellin NK (2011). The IL-17 pathway as a major therapeutic target in autoimmune diseases. Ann N Y Acad Sci.

[CR57] Hueber W, Sands BE, Lewitzky S (2012). Secukinumab, a human anti-IL-17A monoclonal antibody, for moderate to severe Crohn’s disease: unexpected results of a randomised, double-blind placebo-controlled trial. Gut.

[CR58] Huh JR, Littman DR (2012). Small molecule inhibitors of RORγt: targeting Th17 cells and other applications. Eur J Immunol.

[CR59] Huh JR, Leung MW, Huang P (2011). Digoxin and its derivatives suppress Th17 cell differentiation by antagonizing RORγt activity. Nature.

[CR60] Huizinga TW, Fleischmann RM, Jasson M (2014). Sarilumab, a fully human monoclonal antibody against IL-6Rα in patients with rheumatoid arthritis and an inadequate response to methotrexate: efficacy and safety results from the randomised SARIL-RA-MOBILITY Part A trial. Ann Rheum Dis.

[CR61] Ito H, Yamada H, Shibata TN (2011). Dual role of interleukin-17 in pannus growth and osteoclastogenesis in rheumatoid arthritis. Arthritis Res Ther.

[CR62] Jiao Z, Hua S, Wang W (2013). Increased circulating myeloid-derived suppressor cells correlated negatively with Th17 cells in patients with rheumatoid arthritis. Scand J Rheumatol.

[CR63] Kagami S, Rizzo HL, Lee JJ (2010). Circulating Th17, Th22, and Th1 cells are increased in psoriasis. J Invest Dermatol.

[CR64] Kang KY, Kim HO, Kwok SK (2011). Impact of interleukin-21 in the pathogenesis of primary Sjögren’s syndrome: increased serum levels of interleukin-21 and its expression in the labial salivary glands. Arthritis Res Ther.

[CR65] Kasper LH, Everitt D, Leist TP (2006). A phase I trial of an interleukin-12/23 monoclonal antibody in relapsing multiple sclerosis. Curr Med Res Opin.

[CR66] Katsifis GE, Rekka S, Moutsopoulos NM (2009). Systemic and local interleukin-17 and linked cytokines associated with Sjögren’s syndrome immunopathogenesis. Am J Pathol.

[CR67] Kauffman CL, Aria N, Toichi E (2004). A phase I study evaluating the safety, pharmacokinetics, and clinical response of a human IL-12 p40 antibody in subjects with plaque psoriasis. J Invest Dermatol.

[CR68] Kebir H, Kreymborg K, Ifergan I (2007). Human TH17 lymphocytes promote blood-brain barrier disruption and central nervous system inflammation. Nat Med.

[CR69] Kehlen A, Thiele K, Riemann D (2002). Expression, modulation and signalling of IL-17 receptor in fibroblast-like synoviocytes of patients with rheumatoid arthritis. Clin Exp Immunol.

[CR70] Kim HR, Rajaiah R, Wu QL (2008). Green tea protects rats against autoimmune arthritis by modulating disease-related immune events. J Nutr.

[CR71] Kim SE, Yoon JS, Kim KH (2012). Increased serum interleukin-17 in Graves’ ophthalmopathy. Graefes Arch Clin Exp Ophthalmol.

[CR72] Kim J, Kang S, Kim J (2013). Elevated levels of T helper 17 cells are associated with disease activity in patients with rheumatoid arthritis. Ann Lab Med.

[CR73] Kirkham BW, Lassere MN, Edmonds JP (2006). Synovial membrane cytokine expression is predictive of joint damage progression in rheumatoid arthritis: a two-year prospective study (the DAMAGE study cohort). Arthritis Rheum.

[CR74] Kivitz A, Olech E, Borofsky M (2014). Subcutaneous tocilizumab vs placebo in combination with disease modifying antirheumatic drugs in patients with rheumatoid arthritis. Arthritis Care Res.

[CR75] Kobayashi T, Okamoto S, Hisamatsu T (2008). IL23 differentially regulates the Th1/Th17 balance in ulcerative colitis and Crohn’s disease. Gut.

[CR76] Koenders MI, Lubberts E, van de Loo FA (2006). Interleukin-17 acts independently of TNF-alpha under arthritic conditions. J Immunol.

[CR77] Kotake S, Udagawa N, Takahashi N (1999). IL-17 in synovial fluids from patients with rheumatoid arthritis is a potent stimulator of osteoclastogenesis. J Clin Invest.

[CR78] Krueger GG, Langley RG, Leonardi C (2007). A human interleukin-12/23 monoclonal antibody for the treatment of psoriasis. N Engl J Med.

[CR79] Kürtüncü M, Tüzün E, Türkoğlu R (2012). Effect of short-term interferon-β treatment on cytokines in multiple sclerosis: significant modulation of IL-17 and IL-23. Cytokine.

[CR80] Kvarnström M, Ydrefors J, Ekerfelt C (2013). Longitudinal interferon-β effects in multiple sclerosis: differential regulation of IL-10 and IL-17A, while no sustained effects on IFN-γ, IL-4 or IL-13. J Neurol Sci.

[CR81] Kwok SK, Park MK, Cho ML (2012). Retinoic acid attenuates rheumatoid inflammation in mice. J Immunol.

[CR82] Kyttaris VC, Zhang Z, Kuchroo VK (2010). Cutting edge: IL-23 receptor deficiency prevents the development of lupus nephritis in C57BL/6-lpr/lpr mice. J Immunol.

[CR83] Langley RG, Papp K, Gottlieb AB (2013). Safety results from a pooled analysis of randomized, controlled phase II and III clinical trials and interim data from an open-label extension trial of the interleukin-12/23 monoclonal antibody, briakinumab, in moderate to severe psoriasis. J Eur Acad Dermatol Venereol.

[CR84] Lemaître PH, Vokaer B, Charbonnier LM (2013). Cyclosporine a drives a th17- and th2-mediated posttransplant obliterative airway disease. Am J Transplant.

[CR85] Leonardi CL, Kimball AB, Papp KA (2008). Efficacy and safety of ustekinumab, a human interleukin-12/23 monoclonal antibody, in patients with psoriasis: 76-week results from a randomised, double-blind, placebo-controlled trial (PHOENIX 1). Lancet.

[CR86] Li Q, Cong B, Shan B (2011). Cholecystokinin octapeptide exerts its therapeutic effects on collagen-induced arthritis by suppressing both inflammatory and Th17 responses. Rheumatol Int.

[CR87] Lina C, Conghua W, Nan L (2011). Combined treatment of etanercept and MTX reverses Th1/Th2, Th17/Treg imbalance in patients with rheumatoid arthritis. J Clin Immunol.

[CR88] Liu X, Yang P, Lin X (2009). Inhibitory effect of Cyclosporin A and corticosteroids on the production of IFN-gamma and IL-17 by T cells in Vogt-Koyanagi-Harada syndrome. Clin Immunol.

[CR89] Liu Y, Ho RC, Mak A (2012). The role of interleukin (IL)-17 in anxiety and depression of patients with rheumatoid arthritis. Int J Rheum Dis.

[CR90] Lowes MA, Kikuchi T, Fuentes-Duculan J (2008). Psoriasis vulgaris lesions contain discrete populations of Th1 and Th17 T cells. J Invest Dermatol.

[CR91] Lu Y, Chen B, Song JH (2013). Eriocalyxin B ameliorates experimental autoimmune encephalomyelitis by suppressing Th1 and Th17 cells. Proc Natl Acad Sci USA.

[CR92] Ma HL, Napierata L, Stedman N (2010). Tumor necrosis factor alpha blockade exacerbates murine psoriasis-like disease by enhancing Th17 function and decreasing expansion of Treg cells. Arthritis Rheum.

[CR93] Maehara T, Moriyama M, Hayashida JN (2012). Selective localization of T helper subsets in labial salivary glands from primary Sjögren’s syndrome patients. Clin Exp Immunol.

[CR94] Maeshima K, Yamaoka K, Kubo S (2012). The JAK inhibitor tofacitinib regulates synovitis through inhibition of interferon-γ and interleukin-17 production by human CD4+ T cells. Arthritis Rheum.

[CR95] Marwaha AK, Crome SQ, Panagiotopoulos C (2010). Cutting edge: Increased IL-17-secreting T cells in children with new-onset type 1 diabetes. J Immunol.

[CR96] Mease PJ, Genovese MC, Greenwald MW (2014). Brodalumab, an anti-IL17RA monoclonal antibody, in psoriatic arthritis. N Engl J Med.

[CR97] Mei Y, Pan F, Gao J (2011). Increased serum IL-17 and IL-23 in the patient with ankylosing spondylitis. Clin Rheumatol.

[CR98] Meloni F, Solari N, Cavagna L (2009). Frequency of Th1, Th2 and Th17 producing T lymphocytes in bronchoalveolar lavage of patients with systemic sclerosis. Clin Exp Rheumatol.

[CR99] Metawi SA, Abbas D, Kamal MM (2011). Serum and synovial fluid levels of interleukin-17 in correlation with disease activity in patients with RA. Clin Rheumatol.

[CR100] Mitoma H, Horiuchi T, Kimoto Y (2005). Decreased expression of interleukin-21 receptor on peripheral B lymphocytes in systemic lupus erythematosus. Int J Mol Med.

[CR101] Moon YM, Yoon BY, Her YM (2012). IL-32 and IL-17 interact and have the potential to aggravate osteoclastogenesis in rheumatoid arthritis. Arthritis Res Ther.

[CR102] Moon SJ, Park JS, Woo YJ (2014). Rebamipide suppresses collagen-induced arthritis through reciprocal regulation of Th17/Treg cell differentiation and heme oxygenase 1 induction. Arthritis Rheumatol.

[CR103] Mosmann TR, Coffman RL (1989). TH1 and TH2 cells: different patterns of lymphokine secretion lead to different functional properties. Annu Rev Immunol.

[CR104] Mosmann TR, Cherwinski H, Bond MW (2005). Two types of murine helper T cell clone. I. Definition according to profiles of lymphokine activities and secreted proteins. 1986. J Immunol.

[CR105] Murata M, Fujimoto M, Matsushita T (2008). Clinical association of serum interleukin-17 levels in systemic sclerosis: is systemic sclerosis a Th17 disease?. J Dermatol Sci.

[CR106] Nakagawa R, Yoshida H, Asakawa M (2011). Pyridone 6, a pan-JAK inhibitor, ameliorates allergic skin inflammation of NC/Nga mice via suppression of Th2 and enhancement of Th17. J Immunol.

[CR107] Nakano K, Higashi T, Hashimoto K (2008). Antagonizing dopamine D1-like receptor inhibits Th17 cell differentiation: preventive and therapeutic effects on experimental autoimmune encephalomyelitis. Biochem Biophys Res Commun.

[CR108] Nakano K, Yamaoka K, Hanami K (2011). Dopamine induces IL-6-dependent IL-17 production via D1-like receptor on CD4 naive T cells and D1-like receptor antagonist SCH-23390 inhibits cartilage destruction in a human rheumatoid arthritis/SCID mouse chimera model. J Immunol.

[CR109] Nanba T, Watanabe M, Inoue N (2009). Increases of the Th1/Th2 cell ratio in severe Hashimoto’s disease and in the proportion of Th17 cells in intrac Graves’ disease. Thyroid.

[CR110] Nielsen OH, Kirman I, Rüdiger N (2003). Upregulation of interleukin-12 and -17 in active inflammatory bowel disease. Scand J Gastroenterol.

[CR111] Nistala K, Moncrieffe H, Newton KR (2008). Interleukin-17-producing T cells are enriched in the joints of children with arthritis, but have a reciprocal relationship to regulatory T cell numbers. Arthritis Rheum.

[CR112] Nistala K, Adams S, Cambrook H (2010). Th17 plasticity in human autoimmune arthritis is driven by the inflammatory environment. Proc Natl Acad Sci USA.

[CR113] Niu Q, Cai B, Huang ZC (2012). Disturbed Th17/Treg balance in patients with rheumatoid arthritis. Rheumatol Int.

[CR114] Noack M, Miossec P (2014). Th17 and regulatory T cell balance in autoimmune and inflammatory diseases. Autoimmun Rev.

[CR115] Notley CA, Inglis JJ, Alzabin S (2008). Blockade of tumor necrosis factor in collagen-induced arthritis reveals a novel immunoregulatory pathway for Th1 and Th17 cells. J Exp Med.

[CR116] Okamoto Y, Tanaka M, Fukui T (2012). Brazilian propolis inhibits the differentiation of Th17 cells by inhibition of interleukin-6-induced phosphorylation of signal transducer and activator of transcription 3. Immunopharmacol Immunotoxicol.

[CR117] Omoyinmi E, Hamaoui R, Pesenacker A (2012). Th1 and Th17 cell subpopulations are enriched in the peripheral blood of patients with systemic juvenile idiopathic arthritis. Rheumatology.

[CR118] Page G, Miossec P (2005). RANK and RANKL expression as markers of dendritic cell-T cell interactions in paired samples of rheumatoid synovium and lymph nodes. Arthritis Rheum.

[CR119] Pan HF, Leng RX, Feng CC (2013). Expression profiles of Th17 pathway related genes in human systemic lupus erythematosus. Mol Biol Rep.

[CR120] Pan HF, Li XP, Zheng SG (2013). Emerging role of interleukin-22 in autoimmune diseases. Cytokine Growth Factor Rev.

[CR121] Papp KA, Langley RG, Lebwohl M (2008). Efficacy and safety of ustekinumab, a human interleukin-12/23 monoclonal antibody, in patients with psoriasis: 52-week results from a randomised, double-blind, placebo-controlled trial (PHOENIX 2). Lancet.

[CR122] Paris D, Beaulieu-Abdelahad D, Mullan M (2013). Amelioration of experimental autoimmune encephalomyelitis by anatabine. PLoS One.

[CR123] Park JS, Park MK, Lee SY (2012). TWEAK promotes the production of Interleukin-17 in rheumatoid arthritis. Cytokine.

[CR124] Patel DD, Lee DM, Kolbinger F et al (2013) Effect of IL-17A blockade with secukinumab in autoimmune diseases. Ann Rheum Dis 72:iii116–iii123 10.1136/annrheumdis-2012-202371 23253932

[CR125] Pelletier M, Girard D (2007). Biological functions of interleukin-21 and its role in inflammation. ScientificWorldJournal.

[CR126] Pickens SR, Volin MV, Mandelin AM (2010). IL-17 contributes to angiogenesis in rheumatoid arthritis. J Immunol.

[CR127] Piper C, Pesenacker AM, Bending D (2014). Brief report: T cell expression of granulocyte-macrophage colony-stimulating factor in juvenile arthritis is contingent upon Th17 plasticity. Arthritis Rheumatol.

[CR128] Radstake TR, van Bon L, Broen J (2009). The pronounced Th17 profile in systemic sclerosis (SSc) together with intracellular expression of TGFbeta and IFNgamma distinguishes SSc phenotypes. PLoS One.

[CR129] Raza A, Shata MT (2012). Letter: pathogenicity of Th17 cells may differ in ulcerative colitis compared with Crohn’s disease. Aliment Pharmacol Ther.

[CR130] Reich K, Langley RG, Papp KA (2011). A 52-week trial comparing briakinumab with methotrexate in patients with psoriasis. N Engl J Med.

[CR131] Roche JC, Capablo JL, Larrad L (2011). Increased serum interleukin-17 levels in patients with myasthenia gravis. Muscle Nerve.

[CR132] Roşu A, Mărgăritescu C, Stepan A (2012). IL-17 patterns in synovium, serum and synovial fluid from treatment-naïve, early rheumatoid arthritis patients. Rom J Morphol Embryol.

[CR133] Rouvier E, Luciani MF, Mattéi MG (1993). CTLA-8, cloned from an activated T cell, bearing AU-rich messenger RNA instability sequences, and homologousto a herpesvirus saimiri gene. J Immunol.

[CR134] Rovedatti L, Kudo T, Biancheri P (2009). Differential regulation of interleukin 17 and interferon gamma production in inflammatory bowel disease. Gut.

[CR135] Sakai A, Sugawara Y, Kuroishi T (2008). Identification of IL-18 and Th17 cells in salivary glands of patients with Sjögren’s syndrome, and amplification of IL-17-mediated secretion of inflammatory cytokines from salivary gland cells by IL-18. J Immunol.

[CR136] Sakuraba A, Sato T, Kamada N (2009). Th1/Th17 immune response is induced by mesenteric lymph node dendritic cells in Crohn’s disease. Gastroenterology.

[CR137] Samson M, Audia S, Janikashvili N (2012). Brief report: inhibition of interleukin-6 function corrects Th17/Treg cell imbalance in patients with rheumatoid arthritis. Arthritis Rheum.

[CR138] Sandborn WJ, Feagan BG, Fedorak RN (2008). A randomized trial of ustekinumab, a human interleukin-12/23 monoclonal antibody in patients with moderate to severe Crohn’s disease. Gastroenterology.

[CR139] Sandborn WJ, Gasink C, Gao LL (2012). Ustekinumab induction and maintenance therapy in refractory Crohn’s disease. N Engl J Med.

[CR140] Sashinami H, Asano K, Yoshimura S (2012). Salmon proteoglycan suppresses progression of mouse experimental autoimmune encephalomyelitis via regulation of Th17 and Foxp3(+) regulatory T cells. Life Sci.

[CR141] Sato K, Suematsu A, Okamoto K (2006). Th17 functions as an osteoclastogenic helper T cell subset that links T cell activation and bone destruction. J Exp Med.

[CR142] Saxena A, Raychaudhuri SK, Raychaudhuri SP (2011). Interleukin-17-induced proliferation of fibroblast-like synovial cells is mTOR dependent. Arthritis Rheum.

[CR143] Scherl EJ, Kumar S, Warren RU (2010). Review of the safety and efficacy of ustekinumab. Ther Adv Gastroenterol.

[CR144] Schirmer L, Rothhammer V, Hemmer B (2013). Enriched CD161high CCR6 + γδ T cells in the cerebrospinal fluid of patients with multiple sclerosis. JAMA Neurol.

[CR145] Segal BM, Constantinescu C, Raychaudhuri A (2008). Repeated subcutaneous injections of IL-12/23 p40 neutralising antibody, ustekinumab, in patients with relapsing-remitting multiple sclerosis: a phase II, double-blind, placebo-controlled, randomised, dose ranging study. Lancet Neurol.

[CR146] Shah K, Lee WW, Lee SH (2010). Dysregulated balance of Th17 and Th1 cells in systemic lupus erythematosus. Arthritis Res Ther.

[CR147] Shen H, Goodall JC, Hill Gaston JS (2009). Frequency and phenotype of peripheral blood Th17 cells in ankylosing spondylitis and rheumatoid arthritis. Arthritis Rheum.

[CR148] Shi Y, Wang H, Su Z (2010). Differentiation imbalance of Th1/Th17 in peripheral blood mononuclear cells might contribute to pathogenesis of Hashimoto’s thyroiditis. Scand J Immunol.

[CR149] Sieper J, Braun J, Kay J (2014). Sarilumab for the treatment of ankylosing spondylitis: results of a Phase II, randomised, double-blind, placebo-controlled study (ALIGN). Ann Rheum Dis.

[CR150] Smolen JS, Weinblatt ME, Sheng S (2014). Sirukumab, a human anti-interleukin-6 monoclonal antibody: a randomised, 2-part (proof-of-concept and dose-finding), phase II study in patients with active rheumatoid arthritis despite methotrexate therapy. Ann Rheum Dis.

[CR151] Son HJ, Lee J, Lee SY (2014). Metformin attenuates experimental autoimmune arthritis through reciprocal regulation of Th17/Treg balance and osteoclastogenesis. Mediators Inflamm.

[CR152] Souza-Moreira L, Delgado-Maroto V, Morell M (2013). Therapeutic effect of ghrelin in experimental autoimmune encephalomyelitis by inhibiting antigen-specific Th1/Th17 responses and inducing regulatory T cells. Brain Behav Immun.

[CR153] Spuls PI, Hooft L (2012). Brodalumab and ixekizumab, anti-interleukin-17-receptor antibodies for psoriasis: a critical appraisal. Br J Dermatol.

[CR154] Stumhofer JS, Silver J, Hunter CA (2007). Negative regulation of Th17 responses. Semin Immunol.

[CR155] Sugita S, Kawazoe Y, Imai A (2012). Inhibition of Th17 differentiation by anti-TNF-alpha therapy in uveitis patients with Behçet’s disease. Arthritis Res Ther.

[CR156] Tamiya T, Kashiwagi I, Takahashi R (2011). Suppressors of cytokine signaling (SOCS) proteins and JAK/STAT pathways: regulation of T-cell inflammation by SOCS1 and SOCS3. Arterioscler Thromb Vasc Biol.

[CR157] Tanaka Y, Mola EM (2014). IL-6 targeting compared to TNF targeting in rheumatoid arthritis: studies of olokizumab, sarilumab and sirukumab. Ann Rheum Dis.

[CR158] Tanaka M, Okamoto Y, Fukui T (2012). Suppression of interleukin 17 production by Brazilian propolis in mice with collagen-induced arthritis. Inflammopharmacology.

[CR159] Tesmer LA, Lundy SK, Sarkar S (2008). Th17 cells in human disease. Immunol Rev.

[CR160] Tham LS, Tang CC, Choi SL (2014). Population exposure-response model to support dosing evaluation of ixekizumab in patients with chronic plaque psoriasis. J Clin Pharmacol.

[CR161] Toussirot E, Michel F, Béreau M (2013). Ustekinumab in chronic immune-mediated diseases: a review of long term safety and patient improvement. Patient Prefer Adherence.

[CR162] Truchetet ME, Brembilla NC, Montanari E (2011). Increased frequency of circulating Th22 in addition to Th17 and Th2 lymphocytes in systemic sclerosis: association with interstitial lung disease. Arthritis Res Ther.

[CR163] Truchetet ME, Brembilla NC, Montanari E (2013). Interleukin-17A+ cells are increased in systemic sclerosis skin and their number is inversely correlated to the extent of skin involvement. Arthritis Rheum.

[CR164] Tu L, Li S, Fu Y (2012). The therapeutic effects of daphnetin in collagen-induced arthritis involve its regulation of Th17 cells. Int Immunopharmacol.

[CR165] Tuskey A, Behm BW (2014). Profile of ustekinumab and its potential in patients with moderate-to-severe Crohn’s disease. Clin Exp Gastroenterol.

[CR166] Tzellos T, Kyrgidis A, Zouboulis CC (2013). Re-evaluation of the risk for major adverse cardiovascular events in patients treated with anti-IL-12/23 biological agents for chronic plaque psoriasis: a meta-analysis of randomized controlled trials. J Eur Acad Dermatol Venereol.

[CR167] van Hamburg JP, Asmawidjaja PS, Davelaar N (2011). Th17 cells, but not Th1 cells, from patients with early rheumatoid arthritis are potent inducers of matrix metalloproteinases and proinflammatory cytokines upon synovial fibroblast interaction, including autocrine interleukin-17A production. Arthritis Rheum.

[CR168] van Hamburg JP, Asmawidjaja PS, Davelaar N (2012). TNF blockade requires 1,25(OH)2D3 to control human Th17-mediated synovial inflammation. Ann Rheum Dis.

[CR169] van Hamburg JP, Corneth OB, Paulissen SM (2013). IL-17/Th17 mediated synovial inflammation is IL-22 independent. Ann Rheum Dis.

[CR170] Veny M, Esteller M, Ricart E (2010). Late Crohn’s disease patients present an increase in peripheral Th17 cells and cytokine production compared with early patients. Aliment Pharmacol Ther.

[CR171] Wallace KL, Zheng LB, Kanazawa Y (2014). Immunopathology of inflammatory bowel disease. World J Gastroenterol.

[CR172] Wang HH, Dai YQ, Qiu W (2011). Interleukin-17-secreting T cells in neuromyelitis optica and multiple sclerosis during relapse. J Clin Neurosci.

[CR173] Wang J, Ren Z, Xu Y (2012). Epigallocatechin-3-gallate ameliorates experimental autoimmune encephalomyelitis by altering balance among CD4 + T-cell subsets. Am J Pathol.

[CR174] Wen T, Li Y, Wu M (2012). Therapeutic effects of a novel tylophorine analog, NK-007, on collagen-induced arthritis through suppressing tumor necrosis factor α production and Th17 cell differentiation. Arthritis Rheum.

[CR175] Wofford J, Menter A (2014). Ustekinumab for the treatment of psoriatic arthritis. Expert Rev Clin Immunol.

[CR176] Xueyi L, Lina C, Zhenbiao W (2013). Levels of circulating Th17 cells and regulatory T cells in ankylosing spondylitis patients with an inadequate response to anti-TNF-α therapy. J Clin Immunol.

[CR177] Yang J, Chu Y, Yang X (2009). Th17 and natural Treg cell population dynamics in systemic lupus erythematosus. Arthritis Rheum.

[CR178] Yao Z, Painter SL, Fanslow WC (1995). Human IL-17: a novel cytokine derived from T cells. J Immunol.

[CR179] Yoshida H, Kimura A, Fukaya T (2012). Low dose CP-690,550 (tofacitinib), a pan-JAK inhibitor, accelerates the onset of experimental autoimmune encephalomyelitis by potentiating Th17 differentiation. Biochem Biophys Res Commun.

[CR180] Yu B, Guan M, Peng Y (2011). Copy number variations of interleukin-17F, interleukin-21, and interleukin-22 are associated with systemic lupus erythematosus. Arthritis Rheum.

[CR181] Zhang C, Zhang J, Yang B (2008). Cyclosporin A inhibits the production of IL-17 by memory Th17 cells from healthy individuals and patients with rheumatoid arthritis. Cytokine.

[CR182] Zhang L, Li JM, Liu XG (2011). Elevated Th22 cells correlated with Th17 cells in patients with rheumatoid arthritis. J Clin Immunol.

[CR183] Zhang L, Li YG, Li YH (2012). Increased frequencies of Th22 cells as well as Th17 cells in the peripheral blood of patients with ankylosing spondylitis and rheumatoid arthritis. PLoS One.

[CR184] Zhang L, Zhang Y, Zhong W (2014). Heme oxygenase-1 ameliorates dextran sulfate sodium-induced acute murine colitis by regulating Th17/Treg cell balance. J Biol Chem.

[CR185] Zheng L, Ye P, Liu C (2013). The role of the IL-23/IL-17 axis in the pathogenesis of Graves’ disease. Endocr J.

[CR186] Zhou Z, Sun W, Liang Y (2012). Fenofibrate inhibited the differentiation of T helper 17 cells in vitro. PPAR Res.

[CR187] Zivojinovic SM, Pejnovic NN, Sefik-Bukilica MN (2012). Tumor necrosis factor blockade differentially affects innate inflammatory and Th17 cytokines in rheumatoid arthritis. J Rheumatol.

